# BactPepDB: a database of predicted peptides from a exhaustive survey of complete prokaryote genomes

**DOI:** 10.1093/database/bau106

**Published:** 2014-11-05

**Authors:** Julien Rey, Patrick Deschavanne, Pierre Tuffery

**Affiliations:** ^1^INSERM, U973, MTi, F-75205 Paris, France, ^2^Université Paris Diderot, Sorbonne Paris Cité, F-75205 Paris, France and ^3^RPBS, F-75205 Paris, France

## Abstract

With the recent progress in complete genome sequencing, mining the increasing amount of genomic information available should in theory provide the means to discover new classes of peptides. However, annotation pipelines often do not consider small reading frames likely to be expressed. BactPepDB, available online at http://bactpepdb.rpbs.univ-paris-diderot.fr, is a database that aims at providing an exhaustive re-annotation of all complete prokaryotic genomes—chromosomal and plasmid DNA—available in RefSeq for coding sequences ranging between 10 and 80 amino acids. The identified peptides are classified as (i) previously identified in RefSeq, (ii) entity-overlapping (intragenic) or intergenic, and (iii) potential pseudogenes—intergenic sequences corresponding to a portion of a previously annotated larger gene. Additional information is related to homologs within order, predicted signal sequence, transmembrane segments, disulfide bonds, secondary structure, and the existence of a related 3D structure in the Protein Databank. As a result, BactPepDB provides insights about candidate peptides, and provides information about their conservation, together with some of their expected biological/structural features. The BactPepDB interface allows to search for candidate peptides in the database, or to search for peptides similar to a query, according to the multiple properties predicted or related to genomic localization.

**Database URL:** http://www.yeastgenome.org/

## Introduction

Peptides and mini proteins have recently met a regain of interest for therapeutic applications (1, 2). For one part, several breakthroughs have allowed significant progress on some traditional weaknesses of peptides as candidate therapeutics. Chemical modifications among which, to cite some, pegylation (3), stapling (4), glycosilation (5), or the construction of chimeric molecules combining cargos and peptides (6) have shown effective to significantly increase the *in vivo* stability of peptides or their delivery in targeted cells. In addition, the characterization of new classes of peptides offers promising perspectives for development. Antimicrobial peptides are expected to address the urge for the discovery of new antibiotics (7). Bacterial quorum sensing peptides that participate in cell-to-cell communication and bacterial adaptation to specific conditions could also lead to new ways to control bacterial proliferation (8). Peptides extracted from venoms have shown to target very specifically various receptors (9). Cell penetrating peptides raise new promises for the controlled cell-specific penetration of peptides (10).

In general however, the rate of discovery of new peptides remains low. Most frequently, the characterization of natural peptides still relies on cycles of purification and sequencing that prevent large scale exploration. Alternative routes rely on the use of phage display techniques [see for instance (11, 12)] or combinatorial chemical methods (13, 14) that have led to success in the identification of peptides targeting specific protein–protein interactions [e.g. (15)]. Such approaches are however labor-intensive and costly.

Thanks to the progress of high throughput sequencing techniques, an increasing amount of complete genome sequences is becoming available which could constitute an important source for the identification of candidate peptides. Unfortunately, mining this amount of information for the discovery of new active peptides still faces some challenges. First, the identification of candidate genes from genomic sequences cannot be directly related to their effective expression. For eukaryotes, even for expressed genes, the knowledge of genomic sequences does not provide sure information about the expressed sequences, owing to the presence of exons and the fact that peptides such as hormones, for instance, often result from the maturation of preproteins [e.g. (16)]. Prokaryotes do not possess such features to the same extent, and gene identification and chromosomic information should in theory be more straightforward, although not exhaustive, because peptides effectively expressed can be encoded in non-genomic nucleic acid, such as that of plasmids. However, the identification of short coding sequences (SCSs) can be challenging (17), and as a consequence, standard annotation pipelines have shown to identify only few such SCSs (18). Indeed, very few candidate genes of size <50 residues are annotated in the RefSeq database (curated non-redundant sequence database of genomes) (19). Also, in addition to the crude identification of candidates from a single genomic sequence, it also seems desirable to have means to explore if and how much such genes are conserved across species. The more a peptide is conserved, and the more probable is its biological role. For instance, most bacteriocins are genus specific, see (20).

BactPepDB comes as an attempt to organize at a large scale the information available from complete prokaryotic genome sequences. It comes as a complement to more specialized preexisting databases among which databases related to antimicrobial peptides (21–27), predicted secreted bacterial proteins (28), quorum sensing peptides (29), signal peptides (30), anuran peptides (31), peptides including the amino isobutyric acid residues (32), cell penetrating peptides (33) and non-ribosomal peptides (34). It is also distinct from databases such as PepBank (35) or EROP-Moscow (36) that compile information related to peptides acknowledged as biologically active from literature sources, or databases such as PEPX (37) or peptiDB (38) devoted to the structure of protein–peptide complexes. Instead, it compiles information about SCSs of size between 10 and 80 amino acids predicted from the analysis of the complete genome sequence of prokaryotes. For each SCS, different features are predicted to provide information about some of their expected biological/structural features. Finally, the search for homologs within the genomes of each order is performed. Overall BactPepDB proposes a unique and exhaustive survey of candidate peptides over the complete chromosomal information available for prokaryotes, which we believe can be a valuable contribution to assist the identification of new biologically active peptides.

## Construction and Content

### Genome wide identification of the candidate peptides

**Figure 1.**
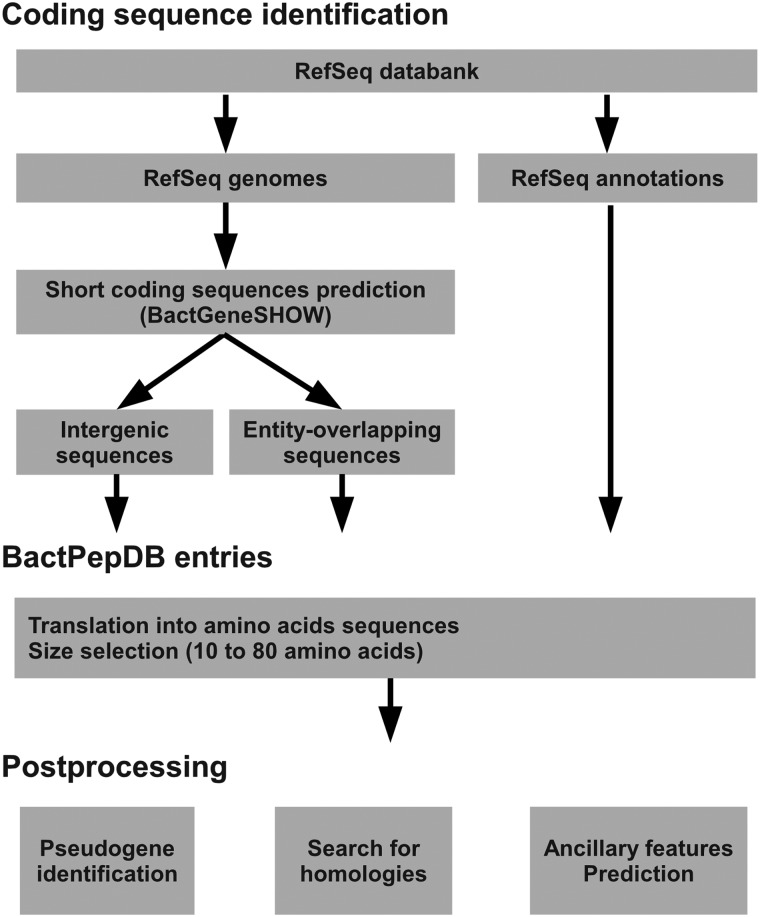
BactPepDB flowchart.

An overview of the processing of BactPepDB is presented in Figure 1. Full genome sequences are collected from RefSeq database (curated non-redundant sequence database of genomes) (19) in FASTA format, together with the organism taxonomy and the corresponding annotations about gene location in the GenBank format. The complete genome and plasmids sequences are then processed using BactgeneSHOW (39), a program specifically designed for the prokaryote genome-wide identification of SCSs. BactgeneSHOW relies on Hidden Markov Models that account for the presence of ribosome-binding sites (RBS) and four types of nucleotide composition of the coding sequences. It analyses both direct and reverse strands. It has been successfully used to identify new SCSs whose expression has been further validated by transcriptomic analyses (17). Predicted SCSs are then translated into amino acid sequences, and only sequences of size between 10 and 80 amino acids are considered for further analysis. Compared to the classical upper limit of peptides of 50 amino acids, the limit of 80 amino acids comes since in some cases, a leader sequence usually on the order of 20–30 amino acids can exist. All such candidate SCSs corresponding to genes already annotated in RefSeq (19) are first identified and inserted in BactPepDB along with their annotations. Then, all the SCSs and RBS genomic coordinates identified by BactgeneSHOW are integrated into the database and the newly detected SCSs are labeled as belonging to intergenic regions or coding regions (entity-overlapping sequences).

### Potential pseudogenes detection

In order to facilitate the identification of false positives, we also perform a search for potential pseudogenes. Pseudogenes are dysfunctional relatives of genes that have lost their protein-coding ability or are otherwise no longer expressed in the cell. Pseudogenes can arise from a partial duplication of active genes and can be found in the intergenic regions. We thus perform a similarity-based approach to detect partially duplicated genes. This approach takes the set of genes detected in intergenic regions of a genome and compares it to the set of RefSeq annotated gene sequences of the same genome. This comparison is accomplished by using the blastp algorithm (40). Cutoff values of 50% identity and 50% coverage of the shortest sequence are set to filter those sequence hits. We recall however that as BactGeneSHOW accounts for the presence of the RBS, all BactPepDB SCSs labeled as potential pseudogenes are possibly expressed.

### Similarity searching

Similarity searching is performed using blastp (40) on the protein sequences. Inter-species sequence conservation inference is achieved by performing an all-against-all search over all the peptide sequences of all the species of an order (as given by organismal taxonomy of RefSeq), considering a sequence identity and coverage of >50% each. Intergenic SCSs are then classified into three different categories according to Warren *et al.* (18). Intergenic SCSs that align to annotated genes from other species are classified as ‘similar to RefSeq entry’ in the genome to which they belong. Intergenic SCSs that align to entity-overlapping sequences from other species are classified as ‘genomic artifacts’. Finally, intergenic SCSs that align to intergenic sequences from other species are labeled as ‘potentially missing’. For these last ones, we require that hit sequence(s) belong to a different taxonomic family. Indeed, a requirement based on different species would not be satisfactory, as there are prokaryotes classified as different species with very similar intergenic sequences due to lack of divergence [for example, *Brucella* species (41)]. Furthermore, the species and genera levels of classification have been shown to be highly variable in prokaryotes (42–45). As such, the next highest taxonomic level (the family) is considered. This requirement is the main evidence used to distinguish sequences that are likely to be real genes from sequences that represent some other conserved elements. In order to give a qualitative estimate of the conservation, two levels of conservation are also provided: a weak conservation stands for the identification of a candidate ortholog in another species of a genus (191 genera), whereas strong conservation stands for the existence of a candidate ortholog in more than two species of a genus, whatever the number of species (105 genera).

### Integration of predicted features

For each candidate peptide, several supplementary analyses are performed and include: (i) the prediction of secondary structure using PsiPred (46), (ii) the prediction of the local conformation prediction as a Structural Alphabet profile (47), (iii) the search for the existence of candidate structure by performing a blastp against the protein data bank (PDB) (48), (iv) the prediction of putative disulfide bonds using DIPro (49), (v) the prediction of transmembrane segments using TMHMM (50), and (vi) the prediction of signal peptides using SignalP (51). Finally, cross references with external databases have also been considered but are presently limited to Bactibase (23).

### Update strategy

Periodical updates of the database are scheduled. Due to the computational cost however, we intend to keep the database up to date on a 6-month basis only.

### Database architecture

BactPepDB was implemented using the combination of the Perl CGI programming language (5.10.1) and a MySQL relational database (5.1.66). The site is running on Apache server (2.2.16-6) installed on a Kernel-based Virtual Machine with Debian 6.0.6 as operating system.

### Database access

BactPepDB is accessible through a web portal at http://bactpepdb.rpbs.univ-paris-diderot.fr. The website requires no authentication.

### Database interface

The search page proposes two possibilities to query BactPepDB. The first is to search for homologs of a peptide sequence among the BactPepDB collection, the second is to search for peptides of BactPepDB matching various criteria. Different parameters can be combined to focus the search: (i) source organism, which represents the sequenced genome the peptides are predicted from, (ii) peptide status, which makes possible to restrict the search to predicted peptides or to peptides already annotated in RefSeq (19), (iii) predicted peptide features, which makes possible to specify information such as the location of the predicted gene (coding or intergenic region), and for peptides in the intergenic regions, if it is a potential pseudogene or not, (iv) sequence features like sequence length or the ability to use regular expressions, and (v) other predicted features such as the presence of a transmembrane segment or a disulfide bond, and peptide conservation across different species of an order (see Similarity Searching section). After running a search, a brief summary of the number of hits matching the different criteria is returned, followed by a list of the hits with the details of the values. This list can be sorted interactively using each criterion as sort key. Finally, links to external resources performing prediction about peptide bioactivity, such as PeptideRanker (52), CAMP (25) or AntiBP2 (53) are proposed. For the similarity search results, pair-wise alignments of the queries with the corresponding hits are shown, but the user also has the possibility to request for a multiple alignment of all the hits using Clustal Omega (54) along with a representation of the residue frequencies at each position produced by WebLogo (55). Each peptide in the database has a unique accession number (e.g. BPDB: 0845925). Subsequently clicking on a BPDB id will lead to the peptide entry content which contains all gathered information concerning this peptide. At any time, users can move back to the search page and narrow their search as search parameters are preserved.

## Results and Discussion

On date of 8 August 2014, the database contains 1 747 413 Peptides from 1226 species (2240 strains including 1598 plasmids) belonging to 557 genera, 218 families and 97 orders. Looking at the agreement between the candidates identified by BactGeneSHOW and the genes previously annotated in RefSeq, we observe that 74% of the SCSs annotated in RefSeq are detected by BactGeneSHOW, in which 66% of the SCSs match exactly, and only 8% differ by either their start or stop positions. The remaining 26% could correspond to SCSs missed by BactGeneSHOW, to SCSs of RefSeq not identified using an automated annotation pipeline and thus possibly having non-typical start codons but observed as expressed by biologists or to genes discarded by the truncature to 80 amino acids. This highlights the limits of a fully automated procedure, but still, a significant amount of information can be retrieved.

**Figure 2.**
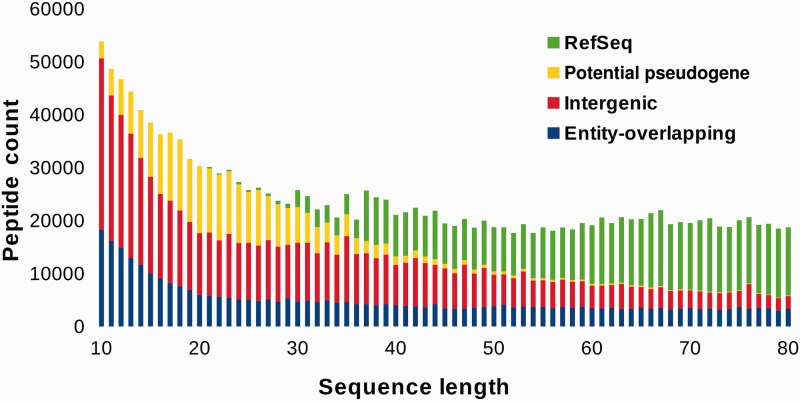
BactPepDB entries according to peptide size.

Among the candidates identified in BactPepDB that are not annotated in RefSeq, one denotes, as illustrated in Figure 2, a clear increase of the number of new entries for smaller SCSs. As a consequence of our flexible definition of potential pseudogenes, the fraction of non-potential pseudogenes new intergenic SCSs for sizes of > 50 amino acids is actually marginal. This suggests that the approach does not identify false positives with high frequencies. Conversely, very few of the candidate sequences identified correspond to already annotated sequences for sizes between 10 and 30 amino acids. Furthermore, one observes some kind of compensation between the increasing number of new intergenic candidates and the decreasing number of RefSeq entries that maintains the number of such peptides rather constant from 80 down to 20 amino acids. Some increase of new intergenic candidates is observed for sizes from 10 to 15 amino acids, which is difficult to interpret at the moment, and remains the subject for further investigation. The fraction of the RefSeq SCSs, the potential pseudogenes peptides, the intra and intergenic SCSs are reported in Table 1, using a three class separation depending on peptide size, to distinguish regions in which new intergenic SCSs are, roughly, predominant, equal and marginal compared to previous RefSeq entries. Overall, SCSs not previously annotated in RefSeq correspond to 70% of BactPepDB. Among these, over 263 000 (15%) are predicted to have disulfide bonds, over 173 000 (10%) to have transmembrane segments and over 30 000 (1.7%) to have signal peptides. Structural homologs of the PDB could be detected for over 100 000 new SCSs.

**Table 1.** BactPepDB entries by categories

	Small	Medium	Large
RefSeq	3946 (0.6)	143 157 (30.4)	362 395 (63.2)
Potential pseudogenes	201 228 (28.6)	51 752 (11.0)	11 743 (2.1)
Intergenic	324 533 (46.2)	189 573 (40.2)	121 897 (21.3)
Entity-overlapping	173 270 (24.6)	86 907 (18.4)	77 012 (13.4)

The three categories correspond to small (<30 amino acids), medium (30–50 amino acids) and large (>50 amino acids) peptide sizes. Peptides already annotated in RefSeq are distinguished from newcomers of BactPepDB categorized as potential pseudogenes, intergenic and entity-overlapping. Fractions in % within brackets.

Considering the intra-genus and intra-order conservation, we observe that close to 184 000 new SCSs detected in the intergenic regions are conserved to some extent across different species of a genus, whereas 112 000 of them are conserved across different taxonomic families of an order. Table 2 presents a comparison of the fraction of the conserved SCSs for these new intergenic SCSs and those preexisting in RefSeq. Interestingly, the fraction of intergenic SCSs that are conserved is similar to that of RefSeq, which suggests that the information of these newcomers is consistent with preexisting one. The fraction of the conserved peptides identified in the intergenic regions appears stable depending of peptide size when considering the different species of a genus (intra-genus) or the different families of an order (intra-order). Overall, depending on the taxonomic level chosen as a requirement for conservation significance, at least 18% of the new intergenic SCSs are conserved.

**Table 2.** Conserved SCSs

		Small	Medium	Large
Intra-genus	RefSeq	978 (25)	40 913 (29)	152 429 (42)
	New intergenic SCSs	84 276 (26)	57 584 (30)	41 447 (34)
Intra-order	RefSeq	750 (19)	20 041 (14)	60 194 (17)
	New intergenic SCSs	61 662 (19)	28 047 (15)	21 940 (18)

Numbers and fractions (% within brackets) of peptide entries that are conserved across species of a genus (intra-genus) and across families of an order (intra-order). Fractions are relative to the total number of entries in each category (see Table 1). The three categories correspond to small (<30 amino acids), medium (30–50 amino acids) and large (>50 amino acids) peptide sizes. Peptides already annotated in RefSeq are distinguished from the new intergenic SCSs of BactPepDB.

### Evaluation of predictions

As the database contains predicted candidates, it is important to assess how likely it can assist the identification of truly expressed peptides. A way to assess BactPepDB-added value comes from experimental studies focusing on specific genomes. For instance, 14 CDSs that were missing from the initial annotation of *Vibrio splendidus LGP32* were recently uncovered (57). We found that 12 of our predictions overlap these missing CDSs. To assess this on a larger scale, we have also compared two versions of BactPepDB based on two versions of the RefSeq database (on date of 11 June 2013 and 30 September 2013) and have found that 125 newly annotated peptides of size comprised between 10 and 80 amino acids were added for genomes that are common to both versions, 33 of which are not of the ‘predicted’ kind and were biologically confirmed. BactGeneSHOW had correctly predicted 89 of these newcomers in the previous version of BactPepDB, among which 24 are now biologically confirmed, which means that about 70% of those newly annotated peptides were already present in BactPepDB before making it to the RefSeq database. Among those predicted peptides, 83 are conserved across different species and only six were unique in their respective order, supporting that peptide conservation is a good measure of peptide expression likeliness.

As the core of BactPepDB relies on BactGeneSHOW, we have also run other gene prediction programs over these genomes to assess BactGeneSHOW performance. GeneMarkHMM 2.6 was able to retrieve 94 of these newcomers, whereas Prodigal 2.5 could only find 38 of them. Although GeneMarkHMM 2.6 slightly outperformed BactGeneSHOW, it is important to note that GeneMarkHMM was apparently inefficient for some genomes, for instance in *Flavobaterium psychrophilum JIP02/86* where none of the five newly annotated genes was detected, whereas BactGeneSHOW retrieves them. Indeed, GeneMarkHMM relies on precalculated heuristic models which may not be suitable for all species whereas BactGeneSHOW relies on a self-learning algorithm.

Finally, another important point to assess is the expected proportion of false positives present in the database. Although this is a very difficult question to answer, we recently gained some insight through RNA deep sequencing data, which reveals smaller intergenic transcripts and mRNA extensions. Analysis of new transcripts from *Escherichia coli str. K-12 substr. MG1655* (56, 58) showed that only 74 predicted sequences of BactPepDB were overlapping the 1094 potentially non-coding transcripts (ncRNA) and long 5′-UTR extensions detected in the intergenic regions of *MG1655*. This is interesting enough because only a very small fraction of these 1094 transcribed regions is supposed to code for peptides.

### Searching for Bactibase homologs

We illustrate here the use of BactPepDB to the search for homologs of Bactibase (23). Bactibase is, in our experience, the only database devoted to antimicrobial peptides for which the complete sequence collection could be downloaded. Among the 219 entries, 197 peptide sequences match the condition to have sequences of size between 10 and 80 residues without non-standard amino acids in their sequence. The genomic information (chromosomal and plasmidic) corresponding to the genus/species was present in BactPepDB for 146 of them. However, this condition does not imply the information should be present in BactPepDB since for one part some variation between strains of a species can occur, and for another part, some peptides can result from the cleavage of preproteins larger than 80 residues, thus out of the scope of BactPepDB. A careful inspection of the literature reporting peptide identification for each Bactibase entry showed that peptides not found in BactPepDB correspond to 11 cases for which preproteins are larger than 80 amino acids, and 37 cases for which it was not possible to conclude, owing to the fact that the peptide sequence was not elucidated using genomic information or that it was not possible to conclude between chromosomal or plasmid encoding. As a result, 98 peptides only were clearly in the scope of BactPepDB.

A similarity-based search in BactPepDB—accepting a correct identification for a hit in the same species, and with over 90% identity—led to the identification of 56 of them. RefSeq annotations were only present for 34 cases over 56. Thus BactPepDB was able to infer new knowledge for 22 cases over 98, a gain of 22%. Furthermore, hits at a lesser sequence identity were found for 22 more peptides. BactPepDB was thus able to grab information for 76 peptides over 98. We remind that all strains of a species are not expected to produce all antimicrobial peptides [see for instance (59–62)]. Overall, such results illustrate that the re-annotation of the complete genome using a method specialized for SCSs can have added value, at least as a preliminary step to confront with additional information.

### Comparison with BAGEL, a database of predicted bacteriocins

We have also analysed the consistency of BactPepDB with BAGEL (24), a resource predicting bacteriocins from genomic data, over a collection of 15 genomes of different genera: *Acaryochloris marina MBIC11017*, *Achromobacter xylosoxidans A8*, *Bacillus cereus AH187*, *Bacillus subtilis BSn5*, *Enterobacter cloacae SCF1*, *Escherichia coli W*, *Geobacillus* sp *C56 T3*, *Lactobacillus casei W56*, *Methanococcus voltae A3*, *Mycobacterium tuberculosis H37Rv*, *Mycobacterium tuberculosis RGTB327*, *Streptococcus pneumoniae AP200*, *Streptococcus thermophilus CNRZ1066*, *Vibrio parahaemolyticus RIMD 2210633*, and *Vibrio vulnificus CMCP6*. Over these genomes, BAGEL returned 713 candidates. We also found 395 of these candidates have a size of >80 amino acids. On the 213 remaining candidates, only 89 are common to BAGEL and BactPepDB. Such difference of 124 candidates is not *per se* surprising since BAGEL relies on Glimmer2 to identify candidates, and it does not consider the presence of a RBS when BactGeneSHOW does—one can thus expect BactGeneSHOW to be more stringent. Among the 89 candidates identified by both BAGEL and BactPepDB, 55 are annotated in RefSeq (and in BactPepDB), in which seven are known bacteriocins, the others being hypothetical bacteriocins. None of the remaining 124 candidates proposed by BAGEL is annotated in RefSeq. Thus, accepting the RefSeq annotation as a criterion to validate the candidates—note that not all RefSeq entries are biologically confirmed—we find BactPepDB would propose a more narrow set of candidates, not discarding any true positive.

## Conclusions and Future Directions

BactPepDB is a database of predicted peptides from a exhaustive survey of complete prokaryote genomes. BactGeneSHOW being a generic approach to the search for SCSs, taking into account the complete spectrum of prokaryotes from archaea to bacteria and the diversity of each category, it is expected that due to the variability in start codon and codon usage, some part of the truly expressed SCSs are not detected. Genome coding specificity, particularly that existing for bacteria and archaea, could be integrated in BactGeneSHOW but this remains the subject for further work. In addition, from our analyses, BactPepDB already shows the ability to retrieve a large part of previously annotated biological peptides when in the scope of the database. BactPepDB could be improved in several other directions. At present, this precludes important sources of prokaryotic information such as those with unusual codons, as well as the incomplete genomes available in RefSeq or other databases from which it should be possible to increase the knowledge of the degree of conservation of candidates. Particularly, it could be of interest to add data from the Ensembl Bacteria database (63) as it contains, on average, more strains per species. Another limit is related to the impossibility to detect peptides resulting from the maturation of large proteins, which is presently beyond the scope of BactPepDB.

Accepting these limitations, it remains that BactPepDB appears to contain new knowledge about SCSs compared to previous RefSeq entries. Although, it is difficult to exactly assess the amount of candidate peptides that may be expressed in some physiological conditions, or that may have a biological activity, BactPepDB provides a rather unique panorama of SCSs over the complete collection of genomes available, at the level of individual sequences but also considering their conservation through genera. The close to 18% of BactPepDB newcomers conserved to some extent could be seeds for further investigations. The detection of small peptides being more difficult using biochemical analyses, BactPepDB is thus expected to assist the experimental discovery of new bioactive peptides.

## References

[1] Vlieghe P. Lisowski V. Martinez J.*.* (2010) Synthetic therapeutic peptides: science and market. Drug Discov. Today, 15, 40–56. 19879957 10.1016/j.drudis.2009.10.009

[2] Audie J. Boyd C. (2010) The synergistic use of computation, chemistry and biology to discover novel peptide-based drugs: the time is right. Curr. Pharm. Des., 16, 567–582. 19929848 10.2174/138161210790361425

[3] Pan C.Q. Buxton J.M. Yung S.L.*.* (2006) Design of a long acting peptide functioning as both a glucagon-like peptide-1 receptor agonist and a glucagon receptor antagonist. J. Biol. Chem., 281, 12506–12515. 16505481 10.1074/jbc.M600127200

[4] LaBelle J.L. Katz S.G. Bird G.H.*.* (2012) A stapled BIM peptide overcomes apoptotic resistance in hematologic cancers. J. Clin. Invest., 122, 2018–2031. 22622039 10.1172/JCI46231 PMC3366394

[5] Elmagbari N.O. Egleton R.D. Palian M.M.*.* (2004) Antinociceptive structure-activity studies with enkephalin-based opioid glycopeptides. J. Pharmacol. Exp. Ther., 311, 290–297. 15166257 10.1124/jpet.104.069393

[6] Svensen N. Walton J.G.A. Bradley M. (2012) Peptides for cell-selective drug delivery. Trends Pharmacol. Sci., 33, 186–192. 22424670 10.1016/j.tips.2012.02.002

[7] Hancock R.E.W. Sahl H.-G. (2006) Antimicrobial and host-defense peptides as new anti-infective therapeutic strategies. Nat. Biotechnol., 24, 1551–1557. 17160061 10.1038/nbt1267

[8] Chen G. Swem L.R. Swem D.L.*.* (2011) A strategy for antagonizing quorum sensing. Mol. Cell, 42, 199–209. 21504831 10.1016/j.molcel.2011.04.003 PMC3092643

[9] Vetter I. Davis J.L. Rash L.D.*.* (2011) Venomics: a new paradigm for natural products-based drug discovery. Amino Acids, 40, 15–28. 20177945 10.1007/s00726-010-0516-4

[10] Montrose K. Yang Y. Sun X.*.* (2013) Xentry, a new class of cell-penetrating peptide uniquely equipped for delivery of drugs. Sci Rep., 3, 1661. 23588666 10.1038/srep01661 PMC3627194

[11] Landon L.A. Zou J. Deutscher S.L. (2004) Is phage display technology on target for developing peptide-based cancer drugs? Curr. Drug Discov. Technol., 1, 113–132. 16472251 10.2174/1570163043335108

[12] Yu L. Yu P.S. Mui E.Y.Y. (2009) Phage display screening against a set of targets to establish peptide-based sugar mimetics and molecular docking to predict binding site. Bioorg. Med. Chem., 17, 4825–4832. 19447041 10.1016/j.bmc.2009.03.054

[13] Lam K.S. (1997) Application of combinatorial library methods in cancer research and drug discovery. Anticancer Drug Des., 12, 145–167. 9154108

[14] Marani M.M. Ceron M.C.M. Giudicessi S.L.*.* (2009) Screening of one-bead-one-peptide combinatorial library using red fluorescent dyes. Presence of positive and false positive beads. J. Comb. Chem., 11, 146–150. 19072229 10.1021/cc800145c

[15] Ricklin D. Lambris J.D. (2008) Compstatin: a complement inhibitor on its way to clinical application. Adv. Exp. Med. Biol., 632, 273–292. 19025129 10.1007/978-0-387-78952-1_20 PMC2700864

[16] Seidah N.G. Chrétien M. (1999) Proprotein and prohormone convertases: a family of subtilases generating diverse bioactive polypeptides. Brain Res., 848, 45–62. 10701998 10.1016/s0006-8993(99)01909-5

[17] Ibrahim M. Nicolas P. Bessières P.*.* (2007) A genome-wide survey of short coding sequences in streptococci. Microbiology, 153, 3631–3644. 17975071 10.1099/mic.0.2007/006205-0

[18] Warren A.S. Archuleta J. Feng W.-C.*.* (2010) Missing genes in the annotation of prokaryotic genomes. BMC Bioinformatics, 11, 131. 20230630 10.1186/1471-2105-11-131 PMC3098052

[19] Pruitt K.D. Tatusova T. Maglott D.R. (2007) NCBI reference sequences (RefSeq): a curated non-redundant sequence database of genomes, transcripts and proteins. Nucleic Acids Res., 35, D61–D65. 17130148 10.1093/nar/gkl842 PMC1716718

[20] Vuyst L.D. Avonts L. Neysens P.*.* (2004) The lactobin a and amylovorin L471 encoding genes are identical, and their distribution seems to be restricted to the species *Lactobacillus amylovorus* that is of interest for cereal fermentations. Int. J. Food Microbiol., 90, 93–106. 14672834 10.1016/s0168-1605(03)00298-8

[21] Fjell C.D. Hancock R.E.W. Cherkasov A. (2007) Amper: a database and an automated discovery tool for antimicrobial peptides. Bioinformatics, 23, 1148–1155. 17341497 10.1093/bioinformatics/btm068

[22] Wang G. Li X. Wang Z. (2009) APD2: the updated antimicrobial peptide database and its application in peptide design. Nucleic Acids Res.*,* 37, D933–D937. 18957441 10.1093/nar/gkn823 PMC2686604

[23] Hammami R. Zouhir A. Hamida J.B.*.* (2007) Bactibase: a new web-accessible database for bacteriocin characterization. BMC Microbiol., 7, 89. 17941971 10.1186/1471-2180-7-89 PMC2211298

[24] deJong A. vanHeel A.J. Kok J.*.* (2010) BAGEL2: mining for bacteriocins in genomic data. Nucleic Acids Res., 38, W647–W651. 20462861 10.1093/nar/gkq365 PMC2896169

[25] Thomas S. Karnik S. Barai R.S.*.* (2010) CAMP: a useful resource for research on antimicrobial peptides. Nucleic Acids Res., 38, D774–D780. 19923233 10.1093/nar/gkp1021 PMC2808926

[26] Sundararajan V.S. Gabere M.N. Pretorius A.*.* (2012) DAMPD: a manually curated antimicrobial peptide database. Nucleic Acids Res., 40, D1108–D1112. 22110032 10.1093/nar/gkr1063 PMC3244992

[27] Piotto S.P. Sessa L. Concilio S.*.* (2012) YADAMP: yet another database of antimicrobial peptides. Int. J. Antimicrob. Agents, 39, 346–351. 22325123 10.1016/j.ijantimicag.2011.12.003

[28] Jehl M.-A. Arnold R. Rattei T. (2011) Effective—a database of predicted secreted bacterial proteins. Nucleic Acids Res., 39, D591–D595. 21071416 10.1093/nar/gkq1154 PMC3013723

[29] Wynendaele E. Bronselaer A. Nielandt J.*.* (2013) Quorumpeps database: chemical space, microbial origin and functionality of quorum sensing peptides. Nucleic Acids Res., 41, D655–D659. 23180797 10.1093/nar/gks1137 PMC3531179

[30] Choo K.H. Tan T.W. Ranganathan S. (2005) SPdb—a signal peptide database. BMC Bioinformatics*,* 6, 249. 16221310 10.1186/1471-2105-6-249 PMC1276010

[31] Novković M. Simunić J. Bojović V.*.* (2012) DADP: the database of anuran defense peptides. Bioinformatics, 28, 1406–1407. 22467909 10.1093/bioinformatics/bts141

[32] Whitmore L. Chugh J.K. Snook C.F.*.* (2003) The peptaibol database: a sequence and structure resource. J. Pept. Sci., 9, 663–665. 14658787 10.1002/psc.533

[33] Gautam A. Singh H. Tyagi A. (2012) CPPsite: a curated database of cell penetrating peptides. Database, **2012**: article ID bas015; doi:10.1093/database/bas015. 10.1093/database/bas015 PMC3296953 22403286

[34] Caboche S. Pupin M. Leclère V.*.* (2008) NORINE: a database of nonribosomal peptides. Nucleic Acids Res., 36, D326–D331. 17913739 10.1093/nar/gkm792 PMC2238963

[35] Shtatland T. Guettler D. Kossodo M.*.* (2007) Pepbank—a database of peptides based on sequence text mining and public peptide data sources. BMC Bioinformatics, 8, 280. 17678535 10.1186/1471-2105-8-280 PMC1976427

[36] Zamyatnin A.A. Borchikov A.S. Vladimirov M.G.*.* (2006) The EROP-Moscow oligopeptide database. Nucleic Acids Res., 34, D261–D266. 16381860 10.1093/nar/gkj008 PMC1347371

[37] Vanhee P. Reumers J. Stricher F.*.* (2010) PepX: a structural database of non-redundant protein-peptide complexes. Nucleic Acids Res., 38, D545–D551. 19880386 10.1093/nar/gkp893 PMC2808939

[38] London N. Movshovitz-Attias D. Schueler-Furman O. (2010) The structural basis of peptide-protein binding strategies. Structure, 18, 188–199. 20159464 10.1016/j.str.2009.11.012

[39] Nicolas P. Bize L. Muri F.*.* (2002) Mining *Bacillus subtilis* chromosome heterogeneities using hidden Markov models. Nucleic Acids Res., 30, 1418–1426. 11884641 10.1093/nar/30.6.1418 PMC101363

[40] Altschul S.F. Gish W. Miller W.*.* (1990) Basic local alignment search tool. J. Mol. Biol., 215, 403–410. 2231712 10.1016/S0022-2836(05)80360-2

[41] Wattam A.R. Williams K.P. Snyder E.E.*.* (2009) Analysis of ten *Brucella* genomes reveals evidence for horizontal gene transfer despite a preferred intracellular lifestyle. J. Bacteriol., 191, 3569–3579. 19346311 10.1128/JB.01767-08 PMC2681906

[42] Gevers D. Cohan F.M. Lawrence J.G.*.* (2005) Opinion: re-evaluating prokaryotic species. Nat. Rev. Microbiol., 3, 733–739. 16138101 10.1038/nrmicro1236

[43] Ward D.M. Cohan F.M. Bhaya D.*.* (2008) Genomics, environmental genomics and the issue of microbial species. Heredity (Edinb), 100, 207–219. 17551524 10.1038/sj.hdy.6801011

[44] Konstantinidis K.T. Tiedje J.M. (2005) Towards a genome-based taxonomy for prokaryotes. J. Bacteriol., 187, 6258– 6264. 16159757 10.1128/JB.187.18.6258-6264.2005 PMC1236649

[45] Konstantinidis K.T. Tiedje J.M. (2005) Genomic insights that advance the species definition for prokaryotes. Proc. Natl. Acad. Sci. U. S. A., 102, 2567–2572. 15701695 10.1073/pnas.0409727102 PMC549018

[46] Jones D.T. (1999) Protein secondary structure prediction based on position-specific scoring matrices. J. Mol. Biol., 292, 195–202. 10493868 10.1006/jmbi.1999.3091

[47] Camproux A.C. Gautier R. Tufféry P. (2004) A Hidden Markov Model derived structural alphabet for proteins. J. Mol. Biol., 339, 591–605. 15147844 10.1016/j.jmb.2004.04.005

[48] Berman H.M. Westbrook J. Feng Z.*.* (2000) The protein data bank. Nucleic Acids Res., 28, 235–242. 10592235 10.1093/nar/28.1.235 PMC102472

[49] Cheng J. Saigo H. Baldi P. (2006) Large-scale prediction of disulphide bridges using kernel methods, two-dimensional recursive neural networks, and weighted graph matching. Proteins, 62, 617–629. 16320312 10.1002/prot.20787

[50] Krogh A. Larsson B. vonHeijne G.*.* (2001) Predicting transmembrane protein topology with a Hidden Markov Model: application to complete genomes. J. Mol. Biol., 305, 567–580. 11152613 10.1006/jmbi.2000.4315

[51] Petersen T.N. Brunak S. vonHeijne G.*.* (2011) SignalP 4.0: discriminating signal peptides from transmembrane regions. Nat. Methods, 8, 785–786. 21959131 10.1038/nmeth.1701

[52] Mooney C. Haslam N.J. Pollastri G.*.* (2012) Towards the improved discovery and design of functional peptides: common features of diverse classes permit generalized prediction of bioactivity. PLoS One, 7, e45012. 23056189 10.1371/journal.pone.0045012 PMC3466233

[53] Lata S. Mishra N.K. Raghava G.P.S. (2010) AntiBP2: improved version of antibacterial peptide prediction. BMC Bioinformatics, 11, S19. 20122190 10.1186/1471-2105-11-S1-S19 PMC3009489

[54] Sievers F. Wilm A. Dineen D.*.* (2011) Fast, scalable generation of high-quality protein multiple sequence alignments using Clustal Omega. Mol. Syst. Biol., 7, 539. 21988835 10.1038/msb.2011.75 PMC3261699

[55] Crooks G.E. Hon G. Chandonia J.-M.*.* (2004) WebLogo: a sequence logo generator. Genome Res., 14, 1188–1190. 15173120 10.1101/gr.849004 PMC419797

[56] Toffano-Nioche C. Nguyen A.N. Kuchly C.*.* (2012) Transcriptomic profiling of the oyster pathogen Vibrio splendidus opens a window on the evolutionary dynamics of the small RNA repertoire in the Vibrio genus. RNA, 18, 2201–2219. 23097430 10.1261/rna.033324.112 PMC3504672

[57] Raghavan R. Sloan D.B. Ochman H. (2012) Antisense transcription is pervasive but rarely conserved in enteric bacteria. MBio, 3, e000156-12. 10.1128/mBio.00156-12 PMC3419515 22872780

[58] Toffano-Nioche C. Luo Y. Kuchly C.*.* (2013) Detection of non-coding RNA in bacteria and archaea using the DETR’PROK Galaxy pipeline. Methods, 63, 60–65. 23806640 10.1016/j.ymeth.2013.06.003

[59] Stephens S.K. Floriano B. Cathcart D.P.*.* (1998) Molecular analysis of the locus responsible for production of plantaricin S, a two-peptide bacteriocin produced by Lactobacillus plantarum LPCO10. Appl. Environ. Microbiol., 64, 1871–1877. 9572965 10.1128/aem.64.5.1871-1877.1998 PMC106244

[60] Maldonado A. Jiménez-Díaz R. Ruiz-Barba J.L. (2004) Induction of plantaricin production in Lactobacillus plantarum NC8 after coculture with specific gram-positive bacteria is mediated by an autoinduction mechanism. J. Bacteriol., 186, 1556–1564. 14973042 10.1128/JB.186.5.1556-1564.2004 PMC344433

[61] Woodruff W.A. Novak J. Caufield P.W. (1998) Sequence analysis of mutA and mutM genes involved in the biosynthesis of the lantibiotic mutacin II in *Streptococcus mutans*. Gene, 206, 37–43. 9461412 10.1016/s0378-1119(97)00578-7

[62] Ross K.F. Ronson C.W. Tagg J.R. (1993) Isolation and characterization of the lantibiotic salivaricin a and its structural gene sala from *Streptococcus salivarius* 20P3. Appl. Environ. Microbiol., 59, 2014–2021. 8357242 10.1128/aem.59.7.2014-2021.1993 PMC182229

[63] Kersey P.J. Allen J.E. Christensen M.*.* (2013) Ensembl genomes 2013: scaling up access to genome-wide data. Nucleic Acids Res., 42, D546–D552. 24163254 10.1093/nar/gkt979 PMC3965094

