# BactPepDB: a database of predicted peptides from a exhaustive survey of complete prokaryote genomes

**DOI:** 10.1093/database/bau119

**Published:** 2014-12-04

**Authors:** Julien Rey, Patrick Deschavanne, Pierre Tuffery

Database 2014 (2014), doi:10.1093/database/bau106

The abstract for the above article contained the incorrect URL to the BactPepDB database. The correct URL is http://bactpepdb.rpbs.univ-paris-diderot.fr.

The publisher apologizes for this mistake.

